# The accessory papillary muscle with inferior J-waves - peculiarity or hidden danger?

**DOI:** 10.1186/1476-7120-7-50

**Published:** 2009-10-29

**Authors:** James Ker, Lorraine du Toit

**Affiliations:** 1 Department of Physiology, University of Pretoria, Pretoria, South Africa; 2 Department of Forensic Medicine, University of Pretoria, Pretoria, South Africa

## Abstract

Originally described in 1953, today the so-called J-wave is the source of much controversy. As a marker of so-called "early repolarization", this variant has been regarded as a totally benign variant since the 1960's. However, since then a wealth of data have indicated that the J-wave may be a marker of a highly arrhythmogenic substrate with a resultant high risk of sudden cardiac death.

In this case report a case of an accessory papillary muscle with a prominent J-wave is described. This may be the first of many possible cases where papillary muscle variants may be the cause of the J-wave.

## Introduction

In 1953 the so-called "J-wave" was described by dr John Osborn (thus, also called the Osborn wave) [1]. This peculiar electrocardiographic deflection was initially described in experimental hypothermia--today realized as an "injury current" which is the result of the fact that hypothermia increases the epicardial potassium current relative to that in the endocardium during ventricular repolarization--this explains the risk of ventricular fibrillation in hypothermia [1].

However, another peculiar electrocardiographic pattern, known as "early repolarization" has been known for more than 60 years [2]. This electrocardiographic pattern is diverse [3], but all of it's variants have one characteristic in common: The "J-wave"--a characteristic slurring or notching, producing a positive hump, found at the junction of the end of the QRS complex and the beginning of the ST segment [2]. Until recently, this variant was considered benign [4] and epidemiologically is found in 2 to 5% of the population, usually in men, young adults, athletes and dark-skinned persons [2].

However, during the last decade, numerous publications appeared, describing J-waves in men with idiopathic ventricular fibrillation [5-10]. Basic electrophysiology have already suggested a critical role of the J-wave in the pathogenesis of idiopathic ventricular fibrillation [11]. Recently, Nam et al [12] examined the incidence of early repolarization among 1395 controls, representative of the general population, and 15 patients with idiopathic ventricular fibrillation. In these 15 patients with idiopathic ventricular fibrillation all known causes, including the long-and short QT-syndromes, Brugada syndrome and catecholaminergic polymorphic ventricular tachycardia have been excluded and 4 of these 15 patients presented with electrical storm (defined as four or more episodes of ventricular fibrillation in one day). Among the control group the incidence of early repolarization was 3.3% and among the idiopathic ventricular fibrillation group it was a staggering 60% with all four patients with electrical storm having early repolarization [12]. Also recently, is the study by Haïssaguerre [13] who found that 31% of 206 patients who were resuscitated after idiopathic ventricular fibrillation have early repolarization--as shown by the J-wave.

Thus, currently it is thought that early repolarization is not always benign as previously thought and that the J-wave is indicative of a highly arrhythmogenic substrate with a high risk of sudden death in some cases.

In this case report an accessory papillary muscle with inferior J-waves--corresponding to the area of the accessory papillary muscle--are shown. It is possible that this may be a uniquely newly discovered group of patients with J-waves.

## Case report

A case report is presented which clearly demonstrates a J-wave in the inferior lead III on the electrocardiogram. An accessory, third papillary muscle is clearly present on the parasternal, short-axis view--corresponding to the area covered by the inferior lead III. It is suggested that this is a new phenotype of the J-wave--caused by accessory papillary muscles.

A 40-year old caucasian male was referred for a cardiovascular examination by his primary care physician due to a peculiar electrocardiogram. The patient was totally asymptomatic with no previous medical problems and the only previous surgical procedure was an appendicectomy. The patient sought medical advice from his primary care physician on yearly health screening tests as he recently reached the age of 40 years.

The clinical examination did not reveal any abnormalities. The electrocardiogram clearly demonstrated a J-wave in the inferior lead III. In addition, a bifid T-wave was present in lead III, consistent with electrocardiographic stigmata of early repolarization and in leads I and V2 striking ST-elevation was present (see additional file 1).

Echocardiography demonstrated an accessory (third) papillary muscle, clearly visible on both the parasternal long-axis (see additional file 2) and the parasternal short-axis view as a separate structure (see additional file 3 and 4).

## Discussion

Various primary and secondary abnormalities of the ventricular papillary muscles has already been described [14]. These abnormalities include, hemangiomas, solitary hypertrophy, papillary fibroelastoma, inclusion cysts, inflammation in Takayasu's arteritis, isolated infarction, hypoplasia as part of ventricular non-compaction and the description of an octopus shaped papillary muscle, causing mid-ventricular obstruction [14]. A growing number of reports are focusing on the electrocardiographic effects of endoventricular structures [15,16].

As discussed, the J-wave is currently a topic of major interest as it is becoming increasingly plausible that this once benign thought marker of early repolarization may be indicative of a highly arrhythmogenic substrate with a high risk of sudden cardiac death.

As a possible anatomical explanation for the J-wave Boineau raised the possibility that the cause may be deep invagination of Purkinje fibers to the subepicardial level, which will result in increased transmural activation, followed by earlier repolarization [17].

This case report, showing a clear J-wave in the inferior lead III in association with an accessory papillary muscle, may be explained by one of two mechanisms: The accessory papillary muscle may be the endoventricular association of deep invagination of Purkinje fibers or the J-wave may be caused by the accessory papillary muscle itself. In light of the recently described arrhythmogenic associations of the J-wave, as discussed above, this case can be regarded as one with a high risk for idiopathic ventricular fibrillation and thus, sudden cardiac death. The whole spectrum of early repolarization consists of an elevation of the QRS-ST junction (the J-point), ORS notching or slurring (the J wave) and a tall, symmetric T-wave [4,12]. The only other published case report on an accessory papillary muscle with electrocardiographic effects is that of an asymptomatic and healthy 15-year old caucasian girl with prominent U-waves in the inferior leads of the electrocardiogram and an accessory papillary muscle, detected by transthoracic echocardiography [16]. It is quite plausible that the U-waves in that case may also represent repolarization abnormalities as the QRS-ST junction is often involved in repolarization abnormalities [4,12].

Therefore, the diagnostic implication for the echocardiographer is that the echocardiogram, which is an ideal diagnostic modality for the evaluation of endoventricular structures may also be utilized to assess the patient for the risk of idiopathic ventricular fibrillation and thus, sudden cardiac death.

## Competing interests

The authors declare that they have no competing interests.

## Authors' contributions

James Ker and Lorraine du Toit are the sole authors. JK performed the echocardiogram and electrocardiography. LD performed the literature search on the prognostic aspects of the J-wave. Both authors participated in the design of the manuscript. JK wrote the case report. LD wrote the introduction and discussion.

Both authors read and approved the final manuscript.

## Supplementary Material

Additional file 1 **Electrocardiogram depicting J-wave**. This is the 12-lead electrocardiogram, clearly demonstrating the J-wave in lead III. Also note the bifid T-wave and ST-segment elevation in leads I and V1--all possibly caused by the accessory papillary muscle. Click here for file

Additional file 2 **Parasternal, long-axis view**. This is the parasternal, long-axis view. Note the accessory papillary muscle, marked with +. Click here for file

Additional file 3 **Parasternal, short-axis view**. This is the parasternal, short-axis view. The accessory papillary muscle is much clearer demonstrated as a separate structure, marked with +. Click here for file

Additional file 4 **Parasternal, short-axis view**. This is a movie clip from the parasternal, short-axis view, demonstrating the accessory papillary muscle as a separate structure. Click here for file
