# Supplementary figures and images for: The accessory papillary muscle with inferior J-waves - peculiarity or hidden danger?
Source: Cardiovasc Ultrasound. 2009 Oct 29;7:50. doi: 10.1186/1476-7120-7-50 (PMC2774670; doi:10.1186/1476-7120-7-50)

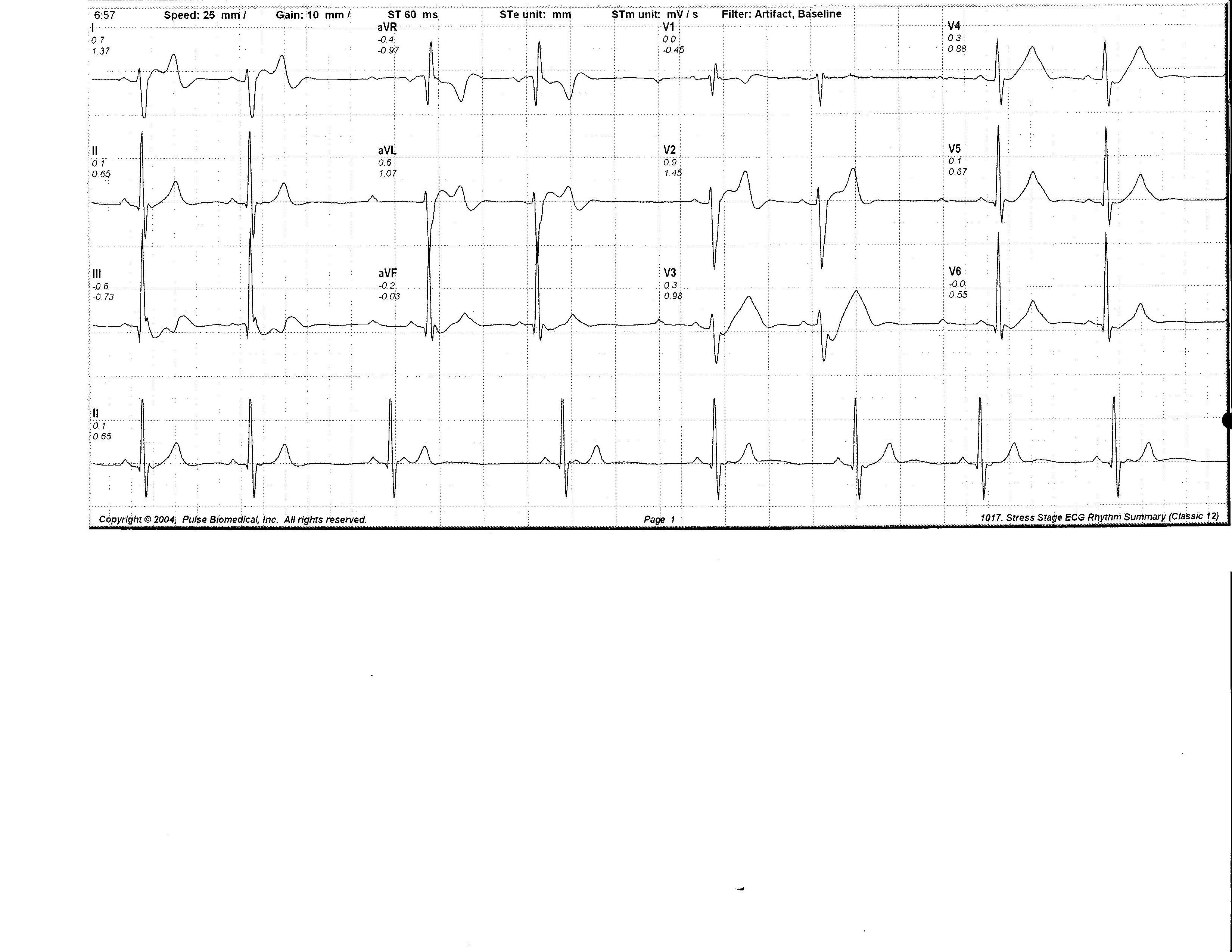

Supplement: Additional file 1 — Electrocardiogram depicting J-wave. This is the 12-lead electrocardiogram, clearly demonstrating the J-wave in lead III. Also note the bifid T-wave and ST-segment elevation in leads I and V1--all possibly caused by the accessory papillary muscle. [file 1476-7120-7-50-S1.jpeg]

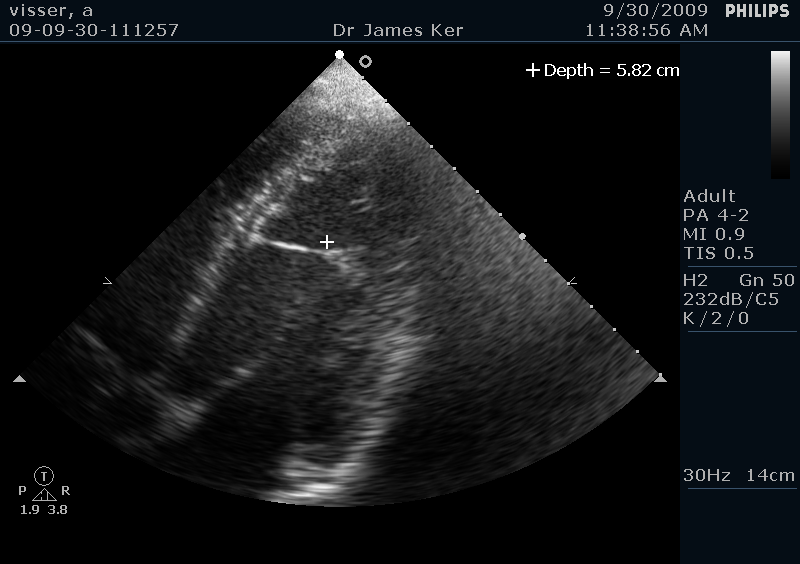

Supplement: Additional file 2 — Parasternal, long-axis view. This is the parasternal, long-axis view. Note the accessory papillary muscle, marked with +. [file 1476-7120-7-50-S2.bmp]

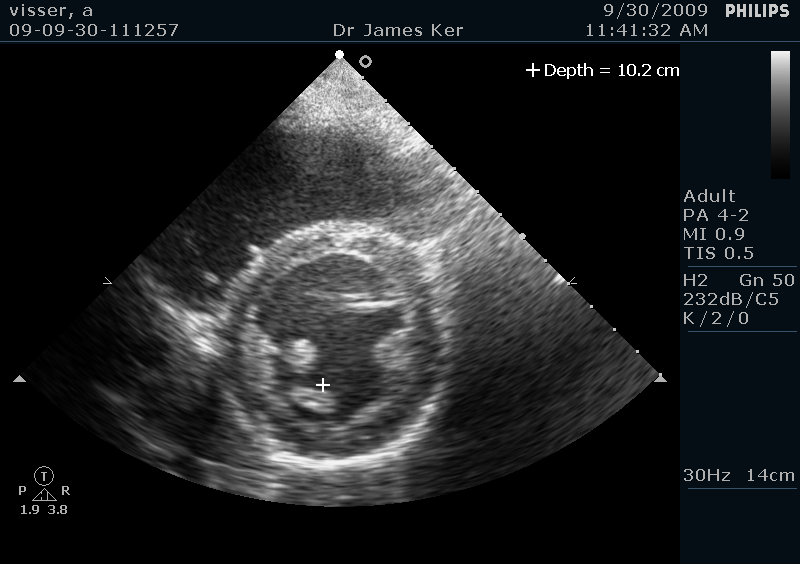

Supplement: Additional file 3 — Parasternal, short-axis view. This is the parasternal, short-axis view. The accessory papillary muscle is much clearer demonstrated as a separate structure, marked with +. [file 1476-7120-7-50-S3.bmp]
